# Native forests but not agroforestry systems preserve arbuscular mycorrhizal fungal species richness in southern Ethiopia

**DOI:** 10.1007/s00572-020-00984-6

**Published:** 2020-09-09

**Authors:** Zerihun Belay, Mesele Negash, Janne Kaseva, Mauritz Vestberg, Helena Kahiluoto

**Affiliations:** 1 School of Applied Natural Science, Adama Science and Technology University, Adama, Ethiopia; 2 Wondo Genet College of Forestry and Natural Resources, Hawassa University, Hawassa, Ethiopia; 3 Natural Resources Institute Finland (Luke), Helsinki, Finland; 4 LUT University, Lappeenranta, Finland

**Keywords:** Agroforestry, *Cordia africana*, Khat, Land-use type, Native forest, Species diversity

## Abstract

The rapid conversion of native forests to farmland in Ethiopia, the cradle of biodiversity, threatens the diversity of the arbuscular mycorrhizal fungi (AMF) pivotal to plant nutrition and carbon sequestration. This study aimed to investigate the impact of this land-use change on the AMF species composition and diversity in southern Ethiopia. Soil samples were collected from nine plots in each of three land-use types: native forest, agroforestry, and khat monocropping. The plots of the three land-use types were located adjacent to each other for each of the nine replicates. Three 10 × 10m subplots per plot were sampled. AMF spores were extracted from the soil samples, spore densities were determined, and species composition and diversity were evaluated through morphological analysis. Both spore density and species richness were statistically significantly higher in the native forest than in the agroforestry plots with no clear difference to khat, whereas the true diversity (exponential of Shannon–Wiener diversity index) did not differ among the three land-use types due to high evenness among the species in agroforestry. In total, 37 AMF morphotypes belonging to 12 genera in Glomeromycota were found, dominated by members of the genera *Acaulospora* and *Glomus*. The highest isolation frequency index (78%) was recorded for *Acaulospora koskei* from native forest. Consequently, the agroforestry system did not appear to aid in preserving the AMF species richness of native forests relative to perennial monocropping, such as khat cultivation. In contrast, the native forest areas can serve as in situ genetic reserves of mycorrhizal symbionts adapted to the local vegetative, edaphic, and microbial conditions.

## Introduction

For several decades in Ethiopia, once the biodiversity cradle of the planet, natural forests have been rapidly converted into farmland. In southern Ethiopia, forests have been converted mainly into agroforestry systems and further into agricultural systems of monocropped perennials such as coffee, pineapple, and eucalyptus, and increasingly to khat (*Catha edulis*), which is used and exported as a stimulant (Abebe 2005; Abebe et al. 2010). This land-use change has resulted in soil degradation, with a decline in soil organic matter (SOM) and nitrogen (Lemenih et al. 2005; Girmay et al. 2008), as well as in changes in other soil physical and chemical properties (Getachew et al. 2012). The devastation to plant diversity in agricultural landscapes is presumed to also affect microbial ecology, especially that of obligatory symbiotic arbuscular mycorrhizal fungi (AMF) (Xiang et al. 2014), which are critical to plant nutrition and soil quality.

AMF are plant-symbiotic soil fungi in the phylum Glomeromycota that are associated with more than 80% of terrestrial plants. The total number of described AMF species was 318 in early October 2019 (www.amf-phylogeny.com). These species belong to 34 genera and 12 families. They play a key role in natural and agricultural ecosystems due to their capacity to form multifunctional arbuscular mycorrhizae (AM), which are considered to be the oldest, most widely distributed, and most important symbiotic associations in nature (Smith and Read 2008). AM increase the soil volume exploited by the host plant, which leads to enhanced plant nutrient uptake (Smith and Read 2008), carbon input to soils (Zhu and Miller 2003), the formation and maintenance of soil structure (Rillig and Mummey 2006), and carbon sequestration (Wang et al. 2016). Other functions of AM include the protection of the root system against biotic and abiotic stresses (de Carvalho et al. 2010). AMF are also a key factor in maintaining plant diversity (Van der Heijden et al. 1998).

AMF diversity and abundance in soil are influenced by vegetation cover (Burrows and Pfleger 2002; Vandenkoornhuyse et al. 2002; Scheublin et al. 2004), soil pH (Alguacil et al. 2016), and soil fertility, particularly phosphorus availability (Kahiluoto et al. 2000; Liu et al. 2016), which is also a determinant of symbiotic AM effectiveness in terms of plant nutrition (Kahiluoto et al. 2001). Tree-based multilayer intercropping (agroforestry) systems are sometimes shown to support a more abundant and diverse AMF community than conventionally managed systems (Chifflot et al. 2009; Lacombe et al. 2009; Bainard et al. 2012; Belay et al. 2015), whereas other studies (e.g., Jefwa et al. 2006) report a lower AMF species diversity in agroforestry systems containing *Sesbania macrantha* and *Sesbania sesban* than in maize monocropping systems. The higher species diversity in the maize fields was suggested to be due to the short maize cropping season, inducing more rapid root dynamics and turnover than in the much longer growth cycles in the agroforestry plots. A higher abundance of AMF spores in agroforestry systems (especially with legume trees) than in monocultures was found in southwestern Ethiopia (Muleta et al. 2008) and in Brazil (Cardoso et al. 2003). The differences in AMF observed in these studies may be a function of the cultivation techniques, climatic variation, or tree–crop combinations used. Evidence from ITS rDNA sequence metadata by Yang et al. (2012) indicated that the distribution of AMF showed high endemism at large scales. This suggests high specificity of AMF for host at different scales (plant taxonomic order and functional group) and high selectivity by host plants for AMF. They suggested that the effects of ecosystemic, biogeographical, continental, and climatic factors on AMF distribution might be mediated by host plants.

We hypothesized that the AMF species diversity and spore density in protected sacred native forests and multilayer agroforestry systems differ from those in monocultured khat farms as a result of the difference in plant diversity, host plants, and soil management. Our specific hypotheses were as follows:

a The land-use type influences the AMF species diversity and richness in accordance with the gradient of the plant diversity and soil management intensity; and
b The impact on the AMF species composition and spore density does not depend on the plant diversity but on the plant species composition.

## Material and methods

### Study sites

The study sites were located in the districts of Wonsho and Shebedino in the Sidama zone, southern Ethiopia, because the three land-use types that represent the typical land-use change continuum in southern Ethiopia are located next to one another in these areas. This enabled the sampling of adjacent fields with similar original site characteristics with known differences in the land-use history as the focus of the study. The study area was located between 7° 00′–7° 06′ northern latitude and 38° 34′–38° 37′ eastern longitude (Asfaw 2003). The agroecology of the study area is characterized by a moist to sub-humid warm subtropical climate with altitudes ranging between 2000 and 2150 m a.s.l. The area has an average annual rainfall of 1200–1500 mm, and the mean monthly temperature is 18–25 °C (Abebe 2013).

This study was undertaken in three land-use types that represent the succession of historical land-use change that has accelerated in recent decades due to population growth and market globalization. The three land-use types, i.e., sacred native forest, agroforestry system, and monocultured perennial khat, have hypothetically distinctive capacities to preserve AMF species diversity and spore density. The native forest patches in agricultural landscapes are remnants of the earlier, more continuous forests that once dominated the area. Later, the forest areas were converted into agricultural land on a large scale in southern Ethiopia, predominantly to multistrata agroforestry systems and recently increasingly to cash monocropping systems such as khat farms. The patches of forest are considered sacred and protected for spiritual reasons, and hence, use of the forests is forbidden and entry to the forest is restricted. Traditional multistrata (i.e., multilayer) agroforestry systems represent the managed ecosystem type providing food security for the local population. Cash crops such as coffee, and later khat, were introduced to the agroforestry systems. The third land-use type considered, khat monocropping, represents the monocropping of perennial cash crops, which has vastly expanded over the past decade. Khat is a cash crop, the production of which has recently rapidly increased due to high-value export demand. Khat monocropping is expected to further expand in East Africa, replacing coffee cultivation, as coffee is more sensitive to climate change.

The native forest is dominated by plant species such as *Cordia africana* Lam, *Afrocarpus falcatus* (*Podocarpus falcatus*) (Thunb.) R. Br. ex Mirb, *Millettia ferruginea* (Hochst.) Baker, *Ficus* spp., *Syzygium guineense* (Willd.) DC., *Aningeria adolfi-friederici*, and *Erythrina bruci* Schweinf. The native dry Afromontane forest converted to multistrata agroforestry and khat farming through gradual harvest of trees selectively and intensification of the land use decades ago. The multistrata agroforestry system included trees, shrubs, and crops grown as integrated layers in the same area. One to three tree species per subplot (subplot refers to a 1 m × 1 m where inventory and soil sampling were conducted) appeared, dominated by *C. africana* and *M. ferruginea*, of the other species, 4% were legumes; the other plants included perennial species such as the food crop enset (*Ensete ventricosum*), the cash crop coffee (*Coffea arabica* L.), and annual food crops such as maize (*Zea mays* L.), yam (*Dioscorea rotundata* L.), taro (*Colocasia esculenta* L.), kale (*Brassica oleracea* L.), and pepper (*Capsicum* spp.). Additionally, khat (*Catha edulis* Forsk.) is often included in the agroforestry systems. The native forest patches called Arosa (2.1 ha) and Abo (32.5 ha) were purposely selected as study sites based on the occurrence of adjacent khat and agroforestry plots. The age of agroforestry management of the sampled agroforestry plots varied from 32 to 54 years, and the khat cultivation history on the khat plots from 15 to 27 years. The khat plant planted, weeded, pruned, and reached to maturity for foliage biomass harvest at the age of 3 years old. Farmers used pesticides for khat plants. The distance between the forest plots and the adjacent agroforestry and khat plots with similar topography ranged from 10 to 100 m.

### Sampling design and soil collection

The soil samples were collected in May–June 2015 using the stratified random sampling method. The basis of stratification was the land-use type. In total, 27 plots (nine for each land-use type) sized 10 m × 10 m and three subplots sized 1 m × 1 m per plot, randomly placed at the corners and center of each plot, were sampled. The samples were collected from 0- to 40-cm depth of soil in triplicate (measurements 1, 2, and 3) in alcohol-sterilized plastic containers, air dried, and stored at room temperature for further analysis. In August 2015, the soil samples arrived at the Natural Resources Institute Finland (Luke), Finland. At Luke in Laukaa, AMF spore densities and species composition were determined with 50 g of replicates 1 and 2 soil samples from a depth of 0–20 cm. Soil physicochemical properties were measured for all three replicates at Luke in Jokioinen, Finland.

### Soil physicochemical characteristics

The soil types of the study sites are mainly classified as Nitisols based on FAO (1988), representing inherently fertile tropical soils with a relatively high nutrient and clay content and deep, permeable structure, often strongly influenced by biological activity. The physicochemical properties of the soil samples are given in Table 1. Soil pH was measured in deionized water (1:2.5 soil:water) (ES ISO 10390), and soil texture was measured with the Bouyoucos hydrometer method (Bouyoucos 1962). Soil plant-available phosphorus was determined by extraction with 0.5 M NaHCO_3_ and measured using Mo-Sb spectrophotometry (Olsen et al. 1954). Total N was determined according to the Kjeldahl method (Bremner and Mulvaney 1982), and total organic carbon was determined by using the method of Walkley and Black (1934).

**Table 1:** Soil physicochemical characteristics of the three land-use types in the Sidama zone, southern Ethiopia

Soil variable	Unit	Native forest	Agroforestry system	Khat monoculture
P_NaHCO3_	ppm	22.1^b^ (14.2, 34.5)	56.4^a^ (36.1, 88.0)	23.4^b^ (15.0, 36.5)
pH	1:2 H_2_O	6.14 ± 0.124	6.43 ± 0.119	6.03 ± 0.210
Total N	%	0.43^a^ ± 0.025	0.29^b^ ± 0.007	0.22^c^ ± 0.017
OC	%	4.98^a^ ± 0.347	3.25^b^ ± 0.048	2.66^c^ ± 0.163
Sand	%	12.0^b^ ± 0.58	26.5^a^ ± 0.50	23.0^a^ ± 2.00
Silt	%	29.5 ± 1.77	24.5 ± 1.77	29.5 ± 1.77
Clay	%	58.5^a^ ± 1.26	49.0^b^ ± 2.38	47.5^b^ ± 0.50

The results represent the means and standard errors of composite samples from nine plots and three subplots for each land-use type (*N* = 27)

*Total N* total nitrogen, *OC* organic carbon, *P*_*NaHCO3*_ NaHCO_3_-extractable phosphorus

The latter was analyzed considering a gamma distribution, and a 95% confidence interval is thus reported. Means followed by the same letter do not differ statistically significantly from each other (*α* = 0.05 followed by the method of Westfall)

### AMF spore analyses

AMF spores were extracted from the soil by wet sieving and decanting (Gerdemann and Nicolson 1963) followed by centrifugation in water and in a 50% sucrose solution (Walker et al. 1982). A 500-μm and a 50-μm sieve were used for wet sieving. After centrifugation, the spores were transferred to a dish of water for examination under a dissecting microscope at magnifications of up to 50 times. Spores were characterized into morphotypes and, whenever possible, identified to species using a high-power microscope. Permanent slides were made using a polyvinyl alcohol-lactoglycerol (PVLG) mountant alone (Omar et al. 1979) or together with Melzer’s reagent (1:1) (Walker et al. 1993).

The Shannon–Wiener index (Krebs 1985) was calculated as a measure of AMF spore diversity (Table 3). The isolation frequency (IF) was calculated as the number of samples in which a given species was isolated as the percentage of the total number of samples. The relative abundance (RA) of spores was calculated as the number of spores of a given AMF species as a percentage of the total number of spores. A sporocarp of the genera *Funneliformis* and *Sclerocystis* was recorded as one spore. The importance value (IV) of an AMF morphotype was used to evaluate the dominance of AMF species based on the IF and RA and was calculated as IV = (IF + RA)/2. IV ≥ 50% indicates that a genus or species is dominant, 10% < IV < 50% applies to common genera or species, and IV ≤ 10% indicates that a genus or species is rare (Chen et al. 2012,2012).

### Statistical analyses

Generalized linear models (GLMs) were used for soil chemical properties, and linear mixed models (LMMs) were used for AMF variables. The soil chemical properties analyzed were normally distributed, except P_NaHCO3_, which was heavily skewed. Thus, the gamma distribution (with a log link) was used for P_NaHCO3_, and estimates were back-transformed to the original scale. The assumption of homogeneous variances was tested with a likelihood ratio test and rejected in all cases except for silt. An information criterion (AIC_C_) was used together with a likelihood ratio test for decision-making. A randomized complete block design (RCBD) was used for the AMF variables and the soil chemical properties of OC and total N, where the nearest three land-use types formed a block. Land use was denoted as a fixed effect, and block was considered a random effect. The mean of three parallel subsamples was calculated to avoid pseudoreplication. All of these variables were analyzed considering a normal distribution, although spore density was log-transformed prior to analysis because of its skewed distribution. The Shannon–Wiener (*H*) is expressed on a logarithmic scale, and to describe the true diversity as the effective number of morphotypes, it needs to be converted to the original scale using Hill number *q* = 1, which is the exponential of *H* (Chao et al. 2014). The model was fitted using the residual maximum likelihood (REML) or residual pseudolikelihood estimation method (RSPL), and the degrees of freedom were calculated using the Kenward–Roger method. The method of Westfall (1997) was used for pairwise comparisons of means, with a significance level of *α* = 0.05. The analyses were performed using the GLIMMIX procedure in SAS Enterprise Guide 7.15 (SAS Institute Inc., Cary, NC, USA).

## Results

### Soil physicochemical parameters

The soil in the study area was slightly acidic to neutral, with mean pH values of 6.1 in native forest, 6.4 in the agroforestry plots, and 6.0 under khat monocropping (Table 1). The soil organic carbon concentration varied between 3.25 and 4.98%, and the soil total nitrogen concentration varied between 0.22 and 0.43%. Statistically significant differences among the three land-use types were found for both carbon and nitrogen concentrations (*p* < 0.05) (Table 1). The P_NaHCO3_ content was significantly higher in the agroforestry plots (56.4 ppm) than in native forest (22.1 ppm) and under khat farming (23.4 ppm) (*p* < 0.05) (Table 1). The native forest plots were more dominated by the clay fraction, while the agroforestry and khat monocrop plots showed a dominant sand fraction.

### AMF spore density

**Fig. 1:**
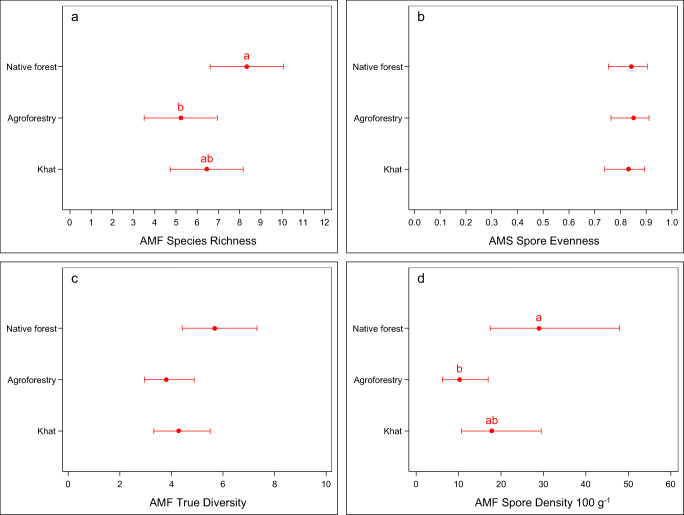
AMF species richness, species evenness, true diversity index value (calculated according to the Shannon–Wiener index using the Hill number of *q* = 1), and spore density (Krebs 1985) of the native forest, multistrata agroforestry, and khat monocropping systems in the Sidama zone, southern Ethiopia. Dot plots show the means and 95% confidence intervals. Means followed by the same letter do not differ statistically significantly from each other (*α* = 0.05 followed by the method of Westfall)

Total spore abundance varied among the three land-use types. The total spore abundance was higher in the native forest (28.9 spores 100-g^−1^ soil) than in the agroforestry plots (10.2 spores 100-g^−1^ soil) (*p* < 0.05) (Fig. 1). The median spore density found under khat monocropping (17.8 spores 100 g^−1^ of soil) did not significantly differ from that in the two other soil types (*α* = 0.05).

### AMF composition

**Fig. 2:**
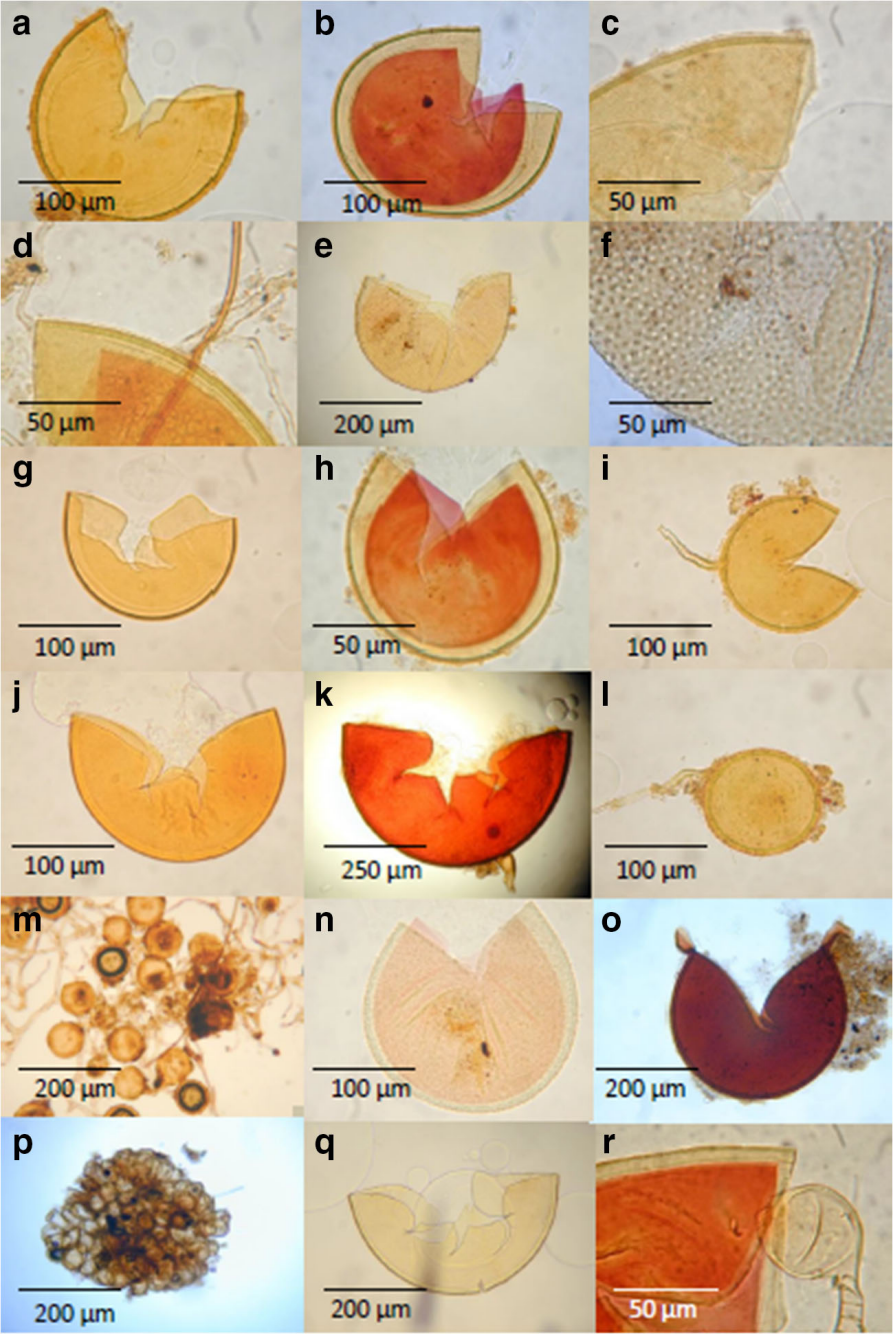
Microscopic photos of some AMF species and morphotypes extracted from soil collected under the three land-use types (native forest, agroforestry system, and khat monocropping system) in Sidama, southern Ethiopia. *Acaulospora* with a smooth spore surface resembling *Acaulospora koskei* stained in polyvinyl alcohol-lactoglycerol (PVLG) (**a**) and in PVLG + Melzer (**b**). *Acaulospora spinosa* in PVLG (**c**) and PVLG + Melzer (**d**). *Acaulospora* sp. 1 with large spores (**e**) having depressions on the surface and a weak Melzer reaction (**f**). A small, yellow *Acaulospora* resembling *A. dilatata* in PVLG (**g**) and PVLG + Melzer (**h**). **i** *Claroideoglomus luteum* in PVLG. **j** *Diversispora epigaea*–like spore in PVLG. **k** Greenish *Gigaspora* sp. 2 with a strong Melzer reaction. **l** *Glomus* sp. 1 with hyphal attachment in PVLG. **m** A clustering, small, thick-walled *Glomus* sp. 4 in PVLG. **n** *Pacispora* sp. in PVLG + Melzer (weak reaction) showing a complicated wall structure with knobs on the outer surface. **o** *Racocetra gregaria*–like spores in PVLG. **p** Sporocarp of a *Sclerocystis* sp. in PVLG. *Scutellospora calospora* in PVLG (**q**) and in PVLG + Melzer (**r**)

Altogether, 37 morphotypes belonging to 12 genera of Glomeromycota were characterized ( Table 2). Twelve (32.4%) and twenty-five (67.6%) morphotypes were identified at the genus and species levels, respectively. Thirteen putative species were members of *Acaulospora*, eight were members of *Glomus*, three each were members of *Claroideoglomus* and *Scutellospora*, and two each were members of *Gigaspora* and *Rhizophagus*. The genera *Cetraspora*, *Diversispora*, *Funneliformis*, *Pacispora*, *Racocetra*, and *Sclerocystis* were each represented by one species. The most frequently found genus was *Acaulospora*, followed by *Glomus*, comprising 36.5% and 21.0% of all species found, respectively (Table 3). Some of the identified species and morphotypes are illustrated in Fig. 2.

**Table 2:** Number of morphotypes per genus characterized in soil sampled from the Sidama zone, southern Ethiopia

Genus	Number of morphotypes characterized
*Acaulospora* Trappe & Gerd (1974)	13
*Cetraspora* Oehl, F.A. Souza & Sieverd. (2009) (2008)	1
*Claroideoglomus* C. Walker & A. Schüßler (2010)	3
*Diversispora* C. Walker & A. Schüßler (2004)	1
*Funneliformis* C. Walker & A. Schüßler (2010)	1
*Gigaspora* Gerd & Trappe (1974)	2
*Glomus* Tul. & C. Tul. (1845)	8
*Pacispora* Sieverd. & Oehl (2004)	1
*Racocetra* Oehl, F.A. Souza & Sieverd. (2008)	1
*Rhizophagus* P.A. Dang. (1896)	2
*Scutellospora* C. Walker & F.E. Sanders (1986)	3
*Sclerocystis* Berk. & Broome (1873)	1
Sum of morphotypes	37

**Table 3:** Distribution of AMF species among land-use types. The AMF species were extracted from the soil of native forest (NF), agroforestry plots (AF), and khat monocropping plots (Kh) in the Sidama zone, southern Ethiopia. Isolation frequency (IF) was calculated as the number of samples in which the given species was isolated as the percentage of the total number of samples. Relative abundance (RA) was calculated as the number of spores of a given AMF species as a percentage of the total number of spores. Importance value (IV) was calculated as IV = (IF + RA)/2

AMF species and morphotypes	IF (%)			RA (%)			IV (%)		
	NF	AF	Kh	NF	AF	Kh	NF	AF	Kh
*Acaulospora dilatata* J.B. Morton (1986)	33.3	0	0	4.9	0	0	19.1	0	0
*Ac. koskei* Błaszk. (1955)	77.8	0	33.3	11.4	0	6.4	44.6	0	19.9
*Ac. spinosa* C. Walker & Trappe (1981)	22.2	0	11.1	3.3	0	2.12	12.8	0	6.6
*Acaulospora* sp. 1. Big spores (± 200 μm), light yellow, depressions at surface	33.3	44.4	33.3	4.9	12	6.4	19.1	28.3	19.9
*Ac.* sp. 2. Big spores (150-200 μm), ash gray, globose, smooth	11.1	0	0	1.6	0	0	6.4	0	0
*Ac.* sp. 3. Big spores, grayish dark brown, smooth	11.1	0	0	1.6	0	0	6.4	0	0
*Ac.* sp. 4. Small spores, light brown, smooth	22.2	0	0	3.3	0	0	12.8	0	0
*Ac.* sp. 5. Yellowish, elongated	11.1	0	0	1.6	0	0	6.4	0	0
*Ac.* sp. 6. Dark brown, hyphae at surface, thick wall	11.1	0	0	1.6	0	0	6.4	0	0
*Ac.* sp. 7. Big spores (180 × 220 μm), yellow-brown, elongated, shiny	22.2	0	0	3.3	0	0	12.8	0	0
*Ac.* sp. 8. Rather big spores, hyaline to white, smooth	22.2	11.1	11.1	3.3	3	2.12	12.8	6.4	6.6
*Ac.* sp. 9. Big spores (200–220 μm), globose, yellow, smooth	11.1	11.1	11.1	1.6	3	2.12	6.4	6.4	6.6
*Ac.* sp. 10. Small- to middle-size spores, yellow, depressions	0	11.1	11.1	0	3	2.12	0	6.4	6.6
*Cetraspora pellucida* Oehl, F.A. & Sieverd. (2009)	11.1	0	11.1	1.6	0	2.12	6.4	0	6.6
*Claroideoglomus claroideum* (N.C. Schenck & G.S. Sm.) C. Walker & A. C (2010)	0	0	11.1	0	0	2.12	0	0	6.6
*C. etunicatum* (W.N. Becker & Gerd.) C. Walker & A. Schüßler (2010)	0	33.3	11.1	0	9	2.12	0	21.2	6.6
*C. luteum* (L.J. Kenn., J.C. Stutz, & J.B. Morton) C. Walker & A. Schüßler (2010)	44.4	33.3	0	6.5	9	0	25.5	21.2	0
*Dentiscutata savannicola* (R.A. Herrera & Ferrer) C. Walker & A. Schüßler (2014)	11.1	0	0	1.6	0	0	6.4	0	0
*Diversispora* sp. C. Walker & A. Schüßler (2004)	33.3	33.3	22.2	4.9	9	4.3	19.1	21.2	13.3
*Funneliformis mosseae* (T.H. Nicolson & Gerd.) C. Walker & A. Schüßler (2010)	0	11.1	44.4	0	3	8.5	0	6.4	26.5
*Gigaspora* sp. 1. Yellowish spores	11.1	0	22.2	1.6	0	4.3	6.4	0	13.3
*Gi.* sp. 2. Spores with a greenish tint	0	11.1	33.3	0	3	6.4	0	6.4	19.9
*Glomus* sp. 1. Brown spores, elongated	22.2	0	0	3.3	0	0	12.8	0	0
*Gl.* sp. 2. Brown spores, globose	33.3	11.1	55.6	4.9	3	10.6	19.1	6.4	33
*Gl.* sp. 3. Big spores, darkly pigmented, irregular	33.3	11.1	0	4.9	3	0	19.1	6.4	0
*Gl.* sp. 4. Small dark brown spores, dense clusters	22.2	0	0	3.3	0	0	12.8	0	0
*Gl.* sp. 5. Big reddish-brown spores (± 190 μm) with thick wall	22.2	11.1	22.2	3.3	3	4.3	12.8	6.4	13.3
*Gl.* sp. 6. Small ash-gray spores	0	0	11.1	0	0	2.12	0	0	6.6
*Gl.* sp. 7. Small yellowish spores	0	11.1	33.3	0	3	6.4	0	6.4	19.9
*Gl.* sp. 8. Big yellowish spores	0	0	11.1	0	0	2.12	0	0	6.6
*Pacispora* sp. Sieverd. & Oehl (2004)	11.1	11.1	66.7	1.6	3	12.8	6.4	6.4	36.5
*Racocetra gregaria* (N.C. Schenck & T.H. Nicolson) Oehl, F.A., Souza & Sieverd. (2008)	22.2	11.1	0	3.3	3	0	12.8	6.4	0
*Rhizophagus fasciculatus* (Thaxt.) C. Walker & A. Schüßler (2010)	0	11.1	0	0	3	0	0	6.4	0
*R. irregularis* (Blaszk., Wubet, Renker, & Buscot) C. Walker & A. Schüßler (2010)	22.2	33.3	22.2	3.3	9	4.3	12.8	21.2	13.3
*Scutellospora calospora* (T.H. Nicolson & Gerd) C. Walker & F.E. Sanders (1986)	44.4	11.1	11.1	6.5	3	2.12	25.5	6.4	6.6
*Scutellospora* sp*.* C. Walker & F.E. Sanders (1986)	44.4	0	0	6.5	0	0	25.5	0	0
*Sclerocystis* sp. Berk. & Broome (1873)	0	44.4	22.2	0	12	4.3	0	28.3	13.3

### Isolation frequency, relative abundance, and dominant AMF species

The genus *Acaulospora* occurred most frequently on average, followed by *Pacispora*, *Diversispora*, and *Claroideoglomus* (Table 3). The highest IF (77.8%) was recorded for *Acaulospora koskei* in native forest, followed by *Pacispora* sp. (67%) and *Glomus* sp. 2 (55.6%) under khat monocropping. The RA of AMF spores was the highest for *Pacispora* sp. (12.8%) under khat monocropping, followed by *Acaulospora* sp. 1 and *Sclerocystis* sp. (12% each) under agroforestry. *Pacispora* sp. was more frequently and abundantly extracted under khat monocropping than under the other land-use types.

According to the IV, no AMF species were dominant in any of the three land-use types (Table 3). However, 18 AMF species (66.6% of the identified species) were regarded as common species in the native forest. Similarly, 6 AMF species (31.6%) under agroforestry and 12 AMF species (54.5%) under khat monocropping were also categorized as common AMF species. In general, compared with that of the other AMF species, the abundance of *Acaulospora koskei* was recorded as high in native forests in terms of the IF, RA, and IV.

### AMF species richness and diversity

Species richness was the highest in the native forest (27 species) and the lowest under agroforestry (19 species) (Table 3). The true diversity (calculated as the exponential of the Shannon–Wiener index) and evenness showed no statistically significant differences among the three land-use types (*p* = 0.070 and *p* = 0.916, respectively). Agroforestry had the lowest index value (3.79), while native forest had the highest index value (5.68). However, agroforestry scored the highest in evenness among the three land-use types (Fig. 1).

## Discussion

The AMF spore densities in native forests were higher than those under agroforestry, whereas the spore densities in khat monocropping systems did not statistically significantly differ from those in the two other land-use types. The relative differences in species richness were similar but smaller, whereas no clear difference in species diversity among the three land-use types could be discerned. Consequently, the hypothesis that land-use type influences the AMF species diversity and richness in accordance with the gradient of the plant diversity and soil management intensity (a) was not fully supported by our data; instead, plant species composition appeared to be as important as plant diversity. Instead, the hypothesis that the impact on the AMF species composition and spore density does not depend on the plant diversity but on the plant species composition (b) was supported by the findings.

### Validity and generalizability

Newly formed spores were lacking in most samples, indicating that the timing of soil collection did not coincide with AMF sporulation. Spores were often damaged or partly decomposed, partly because of the sampling occurring after a relatively moist period. The AMF spore densities in our study varied between 0.12 and 0.35 g^−1^ of soil. These results were somewhat lower than those found by Muleta et al. (2007, 2008) in agroforestry and monocultured coffee systems in southwestern Ethiopia (0.04–1.2-g^−1^ soil) and by Stürmer and Siqueira (2011) in distinct land-use systems in the western Brazilian Amazon (0.51–2.3-g^−1^ soil). However, very high spore densities were reported by Birhane et al. (2018) in dry Afromontane forest in northern Ethiopia (4.5 spores g^−1^ soil) and by Dobo et al. (2016a) in a similar study area and in similar land-use types as in the current study (4.3 to 7.5 g^−1^ of soil). Consequently, the spore densities found here obviously represent those beyond the sporulation period, and the differences among the land-use types reflect relative rather than absolute differences.

The finding of 37 AMF species (morphotypes) belonging to 12 genera of Glomeromycota identified in this study was in the range of previous studies conducted in similar land-use systems in the region. For example, 29 AMF species were identified in Sidama, southern Ethiopia, by Dobo et al. (2016a), and Belay et al. (2013, 2015) found 42 species associated with different land-use types in Ethiopia. However, somewhat lower numbers of AMF species, 12 to 17, were identified by Jefwa et al. (2009, 2012) in soils from indigenous forests to croplands in southern Kenya. Uhlmann et al. (2004) found 44 species in semiarid grasslands in Namibia. Very high numbers of AMF species (60) were detected by Tchabi et al. (2008) in savanna soils from Benin, West Africa, and 61 AMF species were reported by Stürmer and Siqueira (2011) in the Brazilian Amazon. Many morphotypes of the most frequent genera, *Acaulospora* and *Glomus*, could not be identified to the species level, likely because (1) the identification was performed with only field spores, whereas spores of better quality require trap cultures; (2) the samples were collected only during one rainy season (May–June), which induces spore germination and suppresses sporulation, which was perhaps less optimal for obtaining newly formed spores than other seasons (Cuenca and Lovera 2010); and (3) some of the morphotypes might represent new, not yet classified AMF taxa (Oehl et al. 2017).

The lack of clear differences between khat monocropping and the two other land uses should be considered with caution; the growth rhythm and obviously strong influence of khat cultivation on the timing of AMF sporulation reduce the comparability with the other two land uses with many shared plant species. Regarding the clear differences between the native forest and agroforestry plots, the plant species composition and management of the latter are decisive. This draws attention to the importance of the development of agroforestry management to better maintain ecosystem services and soil quality.

### AMF species richness

The differences in AMF species richness are generally determined by plant diversity, density (Burrows and Pfleger 2002; Scheublin et al. 2004; Chifflot et al. 2009) and productivity, land-use intensity (Verbruggen et al. 2010), and soil physicochemical characteristics, including decomposition conditions such as soil moisture and texture. Agroforestry systems are hypothesized to harbor a relatively high AMF species richness and abundance due to the increased species richness and productivity of host plants relative to monocultures (e.g., de Carvalho et al. 2010). However, in the current study, rather lower AMF species richness and spore density were recorded under agroforestry relative to khat monoculture. In addition to the possible confounding differences in the growth rhythm between the agroforestry and khat monoculture systems, a higher plant-available phosphorus supply under agroforestry than in the other land-use types likely decreased the AMF species richness (Xiang et al. 2014) and spore density (Kahiluoto et al. 2001). In contrast to our study, however, Dobo et al. (2016a) reported both increasing spore density and AMF species diversity with an increase in plant-available soil phosphorus in the Sidama zone; a negative influence of very low phosphorus availability (18.4 ppm by Dobo et al. vs. 56.4 ppm NaHCO_3_-extractable phosphorus here) on AM formation and effectiveness has also been previously shown (e.g., Kahiluoto et al. 2000). Rather than by differences in soil properties, the minor or lack of differences between khat monocropping and agroforestry despite the greater plant diversity (Burrows and Pfleger 2002) in agroforestry and higher land-use intensity (Oehl et al. 2010) under khat monocropping may be explained by the difference in host plant species identity (Mathimaran et al. 2007). The maximum (41.71%) mycorrhizal dependency (MD) value was recorded in khat plants when the total MD of crops and agroforestry tree species was evaluated in a greenhouse experiment by Dobo et al. (2016b).

### AMF species composition

One of the causes underlying the dominance of the two genera *Acaulospora* (35.5% of identified species) and *Glomus* (21% of identified species) as also found in other studies in similar agroecosystems (Belay et al. 2013, 2014, 2015; Dobo et al. 2016a), other tropical systems (Stürmer and Siqueira 2011), and Chinese forests (Zhao et al. 2001) as well as in temperate regions (Oehl et al. 2017) is that these genera are the most speciose AMF genera. To date, 50 species have been described from each of these genera in tropical forests (Marinho et al. 2018). The commonness of these genera may also be due to their ability to adapt to different environments, e.g., to tolerate a wide pH range (Marinho et al. 2018) and to produce numerous, often small-diameter spores (Dandan and Zhiwei 2007).

More than 70% of the AMF species identified in this study were specific to one or two of the land-use types. Furthermore, none of the species was dominant based on the IV, even though approximately 29% of the species were common. Only a few species from *Scutellospora*, *Rhizophagus*, *Racocetra*, *Diversispora*, and *Funneliformis* were identified, although these genera are known for their relatively large numbers of species in tropical forests (Marinho et al. 2018). Consistent with this observation, previous studies from Ethiopia and tropical ecosystems have shown that these genera are restricted in their distributions (Stürmer and Siqueira 2011; Belay et al. 2014, 2015).

### AMF spore density

The observation of higher spore densities in natural forest than in agricultural land or monoculture in the current study has also been found in previous studies (Sharmah and Jha 2014). The intensity of land uses such as tillage; mineral fertilization, particularly that related to plant-available phosphorus; and pesticides influences spore abundance (Sharmah and Jha 2014; Dobo et al. 2016b; Stürmer and Siqueira 2011). In contrast to our study, however, Cardoso et al. (2003) and Muleta et al. (2007, 2008) reported higher numbers of spores from agroforestry systems than from monocultured coffee cultivation. This difference in results is obviously due to the difference in the host characteristics and cultivation techniques between coffee and khat (Bainard et al. 2011) and the temporal variation in the number of AMF spores in coffee plantations due to disparity in the phenological stages of the plants (Guadarrama and Alvaarez-Sanchez 1999; Zangaro et al. 2013; Prates Júnior et al. 2019).

### In situ preservation of indigenous AMF

Regarding indigenous vs. introduced AMF (Middleton et al. 2015; Koziol et al. 2018), the most effective species are usually the indigenous AMF adapted to the local plant–soil conditions (Johnson et al. 2010). It has also been shown that management affects ecotype characteristics (Kahiluoto et al. 2000; Oehl et al. 2003). Therefore, in situ preservation of indigenous ecotypes that are adapted to local conditions is very important. Since higher AMF spore density and species richness occurred in native forest, whereas in agroforestry systems they were the lowest (Fig. 1), the native forest patches appear to be invaluable to be protected as reservoirs of the richness of locally adapted, effective AMF species and ecotypes.

## Conclusions

The high density and richness of AMF observed in the protected sacred forest dominated by native plant species highlight the importance of in situ genetic reservoirs of mycorrhizal symbionts in their natural habitats. Such reservoirs might facilitate the introduction of effective and efficient symbionts to agroforestry and agricultural systems restored following mismanagement or being newly managed under sustainable practices where AM have an essential role. Since AMF indigenous to the prevalent soil–plant systems may perform best, with their edaphic origin being especially important (Johnson et al. 2010), such in situ reservoirs are invaluable for soil carbon sequestration, ecosystem restoration, and food security in Ethiopia and, more generally, in East Africa.

## References

[CR1] Abebe T (2005) Diversity in homegarden agroforestry systems of southern Ethiopia. PhD Dissertation, Wageningen University and Research Center, Department of Environmental Sciences

[CR2] Abebe T (2013). Determinants of crop diversity and composition in enset-coffee agroforestry homegardens of southern Ethiopia. J Agric Rural Dev Trop Subtrop.

[CR3] Abebe T, Wiersum KF, Bongers F (2010). Spatial and temporal variation in crop diversity in agroforestry homegardens of southern Ethiopia. Agrof Syst.

[CR4] Alguacil MDM, Torres MP, Montesinos-Navarro A, Roldán A (2016). Soil characteristics driving arbuscular mycorrhizal fungal communities in semiarid Mediterranean soils. Appl Environ Microbiol.

[CR5] Asfaw Z (2003) Tree species diversity, topsoil conditions and arbuscular mycorrhizal association in the Sidama traditional agroforestry land use, southern Ethiopia. PhD Dissertation, Swedish University of Agricultural Sciences

[CR6] Bainard LD, Klironomos JN, Gordon AM (2011). Arbuscular mycorrhizal fungi in tree-based intercropping systems: a review of their abundance and diversity. Pedobiologia.

[CR7] Bainard LD, Koch AM, Gordon AM, Klironomos JN (2012). Temporal and compositional differences of arbuscular mycorrhizal fungal communities in conventional monocropping and tree-based intercropping systems. Soil Biol Biochem.

[CR8] Belay Z, Vestberg V, Assefa F (2013). Diversity and abundance of arbuscular mycorrhizal fungi associated with acacia trees from different land use systems in Ethiopia. Afr J Microbiol Res.

[CR9] Belay Z, Vestberg V, Assefa F (2014). Mycorrhizal status and AMF community structure of fruit crops from low-input cropping system in Showa Robit, Ethiopia. Ethiop J Biol Sci.

[CR10] Belay Z, Vestberg V, Assefa F (2015). Diversity and abundance of arbuscular mycorrhizal fungi across different land use types in a humid low land area of Ethiopia. Trop Subtrop Agroecosyst.

[CR11] Birhane E, Fatumah N, Gidey K, Zenebe A, Mohammed S (2018). Vegetation cover density and disturbance affected arbuscular mycorrhiza fungi spore density and root colonization in a dry Afromontane forest, northern Ethiopia. J For Res.

[CR12] Bouyoucos GJ (1962). Hydrometer method improved for making particle size analysis of soils. Agron J.

[CR13] Bremner JM, Mulvaney CS, Page AL, Miller RH, Keeney DR (1982). Nitrogen-total. Methods of soil analysis. Part 2. 2^nd^ed. Chemical and microbiological properties.

[CR14] Burrows RL, Pfleger FL (2002). Arbuscular mycorrhizal fungi respond to increasing plant diversity. Can J Bot.

[CR15] Cardoso IM, Boddington C, Janssen BH, Oenema O, Kuyper TW (2003). Distribution of mycorrhizal fungal spores in soils under agroforestry and monocultural coffee systems in Brazil. Agroforest Syst.

[CR16] Chao A, Gotelli NJ, Hsieh T (2014). Rarefaction and extrapolation with Hill numbers: a framework for sampling and estimation in species diversity studies. Ecol Monogr.

[CR17] Chen K, Liu WX, Guo SX, Liu RJ, Li M (2012). Diversity of arbuscular mycorrhizal fungi in continuous cropping soils used for pepper production. Afr J Microbiol Res.

[CR18] Chifflot V, Rivest D, Olivier A, Cogliastro A, Khasa D (2009). Molecular analysis of arbuscular mycorrhizal community structure and spores distribution in tree based intercropping and forest systems. Agric Ecosyst Environ.

[CR19] Cuenca G, Lovera M (2010) Seasonal variation and distribution at different soil depths of arbuscular mycorrhizal fungi spores in tropical sclerophyllous shrubland. Botany 88:54–64

[CR20] Dandan Z, Zhiwei Z (2007). Biodiversity of arbuscular mycorrhizal fungi in the hot-dry valley of the Jinsha River, southwest China. Appl Soil Ecol.

[CR21] de Carvalho AMX, Tavares CR, Cardoso IM, Kuyper TW, Dion P (2010). Mycorrhizal associations in agroforestry systems. Soil biology and agriculture in the tropics.

[CR22] Dobo B, Asefa F, Asfaw Z (2016). Diversity and abundance of arbuscular mycorrhizal fungi under different plant and soil properties in Sidama, southern Ethiopia. Agroforest Syst.

[CR23] Dobo B, Asefa F, Asfaw Z (2016). Phosphorus requirement for colonization by arbuscular mycorrhizal fungi (AMF) and effect of AMF inoculants on growth of perennial crops and agroforestry trees. East Afr J Sci.

[CR24] FAO (1988) Soil map of the world. Revised legend, 527 by FAO–UNESCO–ISRIC. World Soil Resources Report No. 60. Rome

[CR25] Gerdemann JW, Nicolson TH (1963). Spores of mycorrhizal *Endogone* species extracted from soil by wet sieving and decanting. Trans Br Mycol Soc.

[CR26] Getachew F, Abdulkadir A, Lemenih M, Fetene A (2012). Effects of different land uses on soil physical and chemical properties in Wondo Genet area, Ethiopia. New York Sc J.

[CR27] Girmay G, Singh BR, Mitiku H, Borresen T, Lal R (2008). Carbon stocks in Ethiopian soils in relation to land use and soil management. Land Degrad Dev.

[CR28] Guadarrama P, Alvaarez-Sanchez FJ (1999). Abundance of arbuscular mycorrhizal fungi spores in different environments in a tropical rain forest, Veracruz, Mexico. Mycorrhiza.

[CR29] Jefwa JM, Sinclair R, Maghembe JA (2006). Diversity of glomale mycorrhizal fungi in maize/sesbania intercrops and maize monocrop systems in southern Malawi. Agrofor Syst.

[CR30] Jefwa JM, Mung’atu J, Okoth P, Muya E, Roimen H, Njuguini S (2009). Influence of land use types on occurrence of arbuscular mycorrhiza fungi in the high altitude regions of Mt. Kenya. Trop Subtrop Agroecosyst.

[CR31] Jefwa JM, Okoth S, Wachira P, Karanja N, Kahindi J, Njuguini S, Ichami S, Mung’atu J, Okoth P, Huising J (2012). Impact of land use types and farming practices on occurrence of arbuscular mycorrhizal fungi (AMF) Taita-Taveta district in Kenya. Agri Ecosyst Environ.

[CR32] Johnson NC, Wilson GWT, Bowker MA, Wilson JA, Miller RM (2010). Resource limitation is a driver of local adaptation in mycorrhizal symbioses. PNAS.

[CR33] Kahiluoto H, Ketoja E, Vestberg M (2000). Promotion of utilization of arbuscular mycorrhiza through reduced P fertilization 1. Bioassays in a growth chamber. Plant Soil.

[CR34] Kahiluoto H, Ketoja E, Vestberg M, Saarela I (2001). Promotion of AM utilization through reduced P fertilization 2. Field studies. Plant Soil.

[CR35] Koziol L, Schultz PA, House GL, Bauer JT, Middleton EL, Bever JD (2018). The plant microbiome and native plant restoration: the example of native mycorrhizal fungi. BioScience.

[CR36] Krebs CJ (1985). Ecology. The experimental analysis of distribution and abundance.

[CR37] Lacombe S, Bradley RL, Hamel C, Beaulieu C (2009). Do tree-based intercropping systems increase the diversity and stability of soil microbial communities?. Agri Ecosyst Environ.

[CR38] Lemenih M, Karltun E, Olsson M (2005). Soil organic matter dynamics after deforestation along a farm field chronosequence in southern highlands of Ethiopia. Agri Ecosyst Environ.

[CR39] Liu W, Zhang Y, Jiang S, Deng Y, Christie P, Murray PJ, Li X, Zhang J (2016) Arbuscular mycorrhizal fungi in soil and roots respond differently to phosphorus inputs in an intensively managed calcareous agricultural soil. Sci Rep 6. 10.1038/srep24902 10.1038/srep24902 PMC4840358 27102357

[CR40] Marinho F, da Silva IR, Oehl F, Maia LC (2018). Checklist of arbuscular mycorrhizal fungi in tropical forests. Sydowia.

[CR41] Mathimaran N, Ruh R, Jama B, Verchot L, Frossard E, Jansa J (2007). Impact of agricultural management on arbuscular mycorrhizal fungal communities in Kenyan ferralsol. Agr Ecosyst Environ.

[CR42] Middleton EL, Richardson S, Koziol L, Palmer CE, Yermakov Z, Henning JA, Schultz PA, Bever JD (2015). Locally adapted arbuscular mycorrhizal fungi improve vigor and resistance to herbivory of native prairie plant species. Ecosphere.

[CR43] Muleta D, Assefa F, Nemomissa S, Granhall U (2007). Composition of coffee shade tree species and density of indigenous arbuscular mycorrhizal fungi (AMF) spores in Bonga natural coffee forest, southwestern Ethiopia. For Ecol Manag.

[CR44] Muleta D, Assefa F, Nemomissa S, Granhall U (2008). Distribution of arbuscular mycorrhizal fungi spores in soil of southwestern Ethiopia. Biol Fertil Soils.

[CR45] Oehl F, Sieverding E, Ineichen K, Mäder P, Boller T, Wiemken A (2003). Impact of land use intensity on the species diversity of arbuscular mycorrhizal fungi in agroecosystems of Central Europe. Appl Environ Microbiol.

[CR46] Oehl F, Laczko E, Bogenrieder A, Stahr K, Bösch R, van der Heijden M, Sieverding E (2010). Soil type and land use intensity determine the composition of arbuscular mycorrhizal fungal communities. Soil Biol Biochem.

[CR47] Oehl F, Laczko E, Oberholzer H, Jansa J, Egli S (2017). Diversity and biogeography of arbuscular mycorrhizal fungi in agricultural soils. Biol Fertil Soils.

[CR48] Olsen SR, Cole, CV, Watanabe FS, Dean L (1954) Estimation of available phosphorus in soils by extraction with sodium bicarbonate. USDA Circular No 939

[CR49] Omar MB, Bolland L, Heather WA (1979). A permanent mounting medium for fungi. Bull Br Mycol Soc.

[CR50] Prates Júnior P, Moreira BC, da Silva MCS, Veloso TGR, Stürmer SL, Fernandes RBA (2019). Agroecological coffee management increases arbuscular mycorrhizal fungi diversity. PLoS One.

[CR51] Rillig MC, Mummey DL (2006). Mycorrhizas and soil structure. New Phytol.

[CR52] Scheublin TR, Ridgway KP, Young JPW, van der Heijden MGA (2004). Non legumes, legumes, and root nodules harbor different arbuscular mycorrhizal fungal communities. Appl Environ Microbiol.

[CR53] Sharmah D, Jha K (2014). Diversity of arbuscular mycorrhizal fungi in undisturbed forest, slash-and-burn field, and monoculture forest of Indo-Burma megadiverse region. Braz J Bot.

[CR54] Smith SE, Read DJ (2008) Mycorrhizal symbiosis, 3rd edn. Elsevier Ltd

[CR55] Stürmer SL, Siqueira JO (2011). Species richness and spore abundance of arbuscular mycorrhizal fungi across distinct land uses in western Brazilian Amazon. Mycorrhiza.

[CR56] Tchabi A, Coyne D, Hountondji F, Lawouin L, Wiemken A, Oehl F (2008). Arbuscular mycorrhizal fungal communities in sub-Saharan savannas of Benin, West Africa, as affected by agricultural land use intensity and ecological zone. Mycorrhiza.

[CR57] Uhlmann E, Görke C, Petersen A, Oberwinkler F (2004). Arbuscular mycorrhizae from semiarid regions of Namibia. Can J Bot.

[CR58] Van der Heijden MGA, Klironomos JN, Ursic M, Moutoglis P, Streitwolf-Engel R, Boller T, Wiemken A, Sanders IR (1998). Mycorrhizal fungal diversity determines plant biodiversity, ecosystem variability and productivity. Nature.

[CR59] Vandenkoornhuyse P, Husband R, Daniell TJ, Watson IJ, Duck JM, Fitter AH, Young JPW (2002). Arbuscular mycorrhizal community composition associated with two plant species in a grassland ecosystem. Mol Ecol.

[CR60] Verbruggen E, Röling WFM, Gamper HA, Kowalchuk GA, Verhoef HA, van der Heijden MGA (2010). Positive effects of organic farming on below-ground mutualists: large-scale comparison of mycorrhizal fungal communities in agricultural soils. New Phytol.

[CR61] Walker C, Mize CW, McNabb HS (1982). Populations of endogonaceous fungi at two locations in Iowa. Can J Bot.

[CR62] Walker C, Gianinazzi-Pearson V, Marion-Espinasse H (1993). *Scutellospora castanea*, a newly described arbuscular mycorrhizal species. Cryptog Mycolog.

[CR63] Walkley A, Black I (1934). An examination of the Degtjareff method for determining organic carbon in soils: effect of variations in digestion conditions and of inorganic soil constituents. Soil Sci.

[CR64] Wang ZG, Bi YL, Jiang B, Zhakypbek Y, Peng SP, Liu WW, Liu H (2016). Arbuscular mycorrhizal fungi enhance soil carbon sequestration in the coalfields, northwest China. Sci Rep.

[CR65] Westfall PH (1997). Multiple testing of general contrasts using logical constraints and correlations. J A Stat Assoc.

[CR66] Xiang D, Verbruggen E, Hu Y, Veresoglou SD, RIllig MC, Zhou W, Xu T, Li H, Hao Z, Chen Y, Chen B (2014). Land use influences arbuscular mycorrhizal fungal communities in the farming–pastoral ecotone of northern China. New Phytol.

[CR67] Yang H, Zang Y, Yuan Y, Tang J, Chen X (2012). Selectivity by host plants affects the distribution of arbuscular mycorrhizal fungi: evidence from ITS rDNA sequence metadata. BMC Evol Biol.

[CR68] Zangaro W, Rostirola LV, de Souza PB, de Almeida AR, Lescano LEAM, Rondina ABL, NagueiraMA CR (2013). Root colonization and spore abundance of arbuscular mycorrhizal fungi in distinct successional stages from an Atlantic rainforest biome in southern Brazil. Mycorrhiza.

[CR69] Zhao ZW, Xia YM, Qin XZ, Li XW, Cheng LZ, Sha T, Wang GH (2001) Arbuscular mycorrhizal status of plants and the spore density of arbuscular mycorrhizal fungi in the tropical rain forest of Xishuangbanna, southwest China. Mycorrhiza 11:159–162 10.1007/s005720100117 24595436

[CR70] Zhu YG, Miller RM (2003). Carbon cycling by arbuscular mycorrhizal fungi in soil-plant systems. Trends Plant Sci.

